# Supplementary figures and images for: Genomic, functional and structural analyses elucidate evolutionary innovation within the sea anemone 8 toxin family
Source: BMC Biol. 2023 May 24;21:121. doi: 10.1186/s12915-023-01617-y (PMC10210398; doi:10.1186/s12915-023-01617-y)

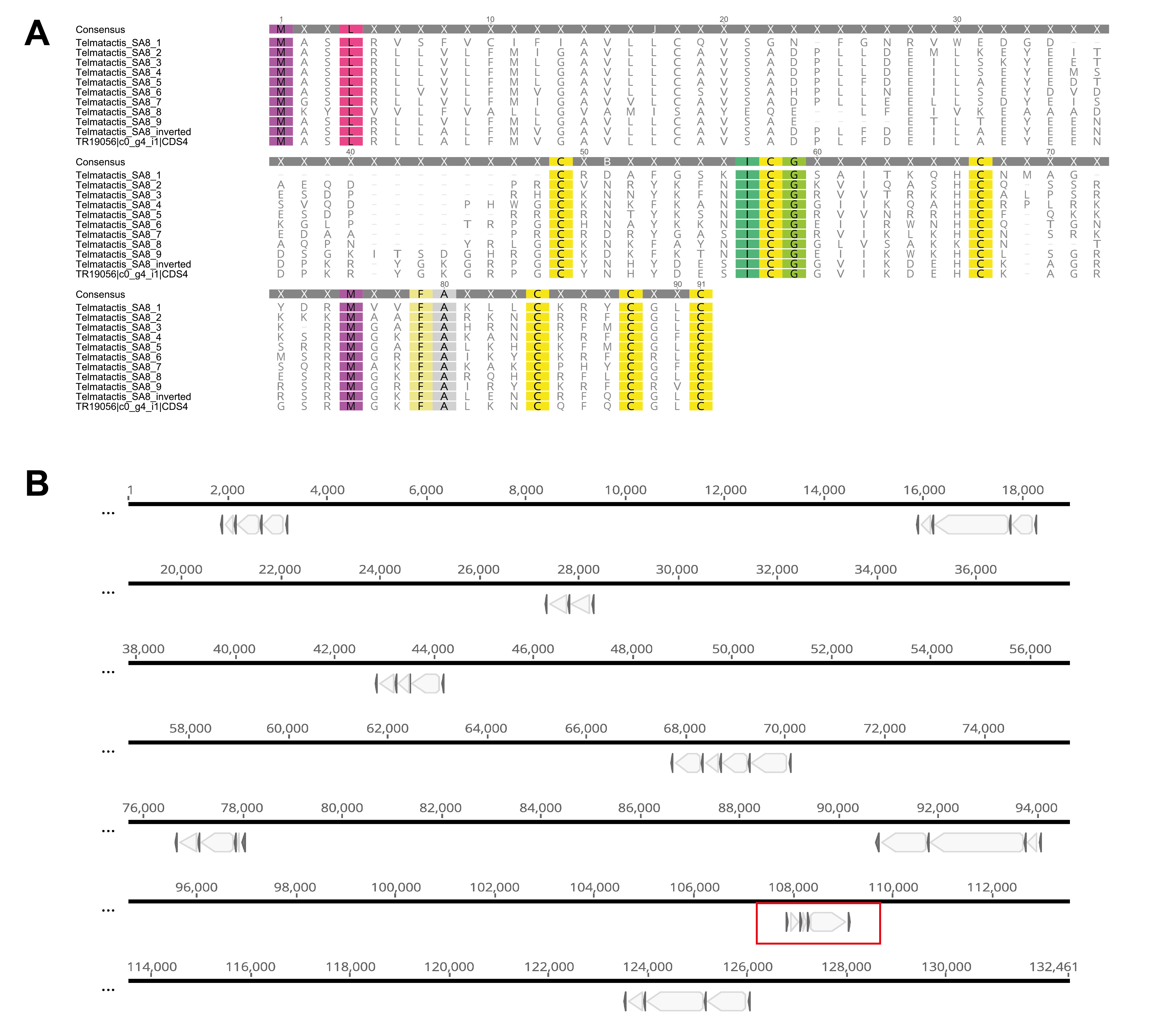

Supplement: Supplementary file 4 — Additional file 4: Figure S1. Sequence and structure of T. stephensoni SA8 sequences. Alignment of SA8 gene and peptide sequences from T. stephensoni, with conserved cysteine framework, glycine residue, and FA dyad highlighted. The nine clustered genes of T. stephensoni are composed of 3–5 microexons and large introns. The inverted T. stephensoni SA8 gene is indicated in red. [file 12915_2023_1617_MOESM4_ESM.tif]

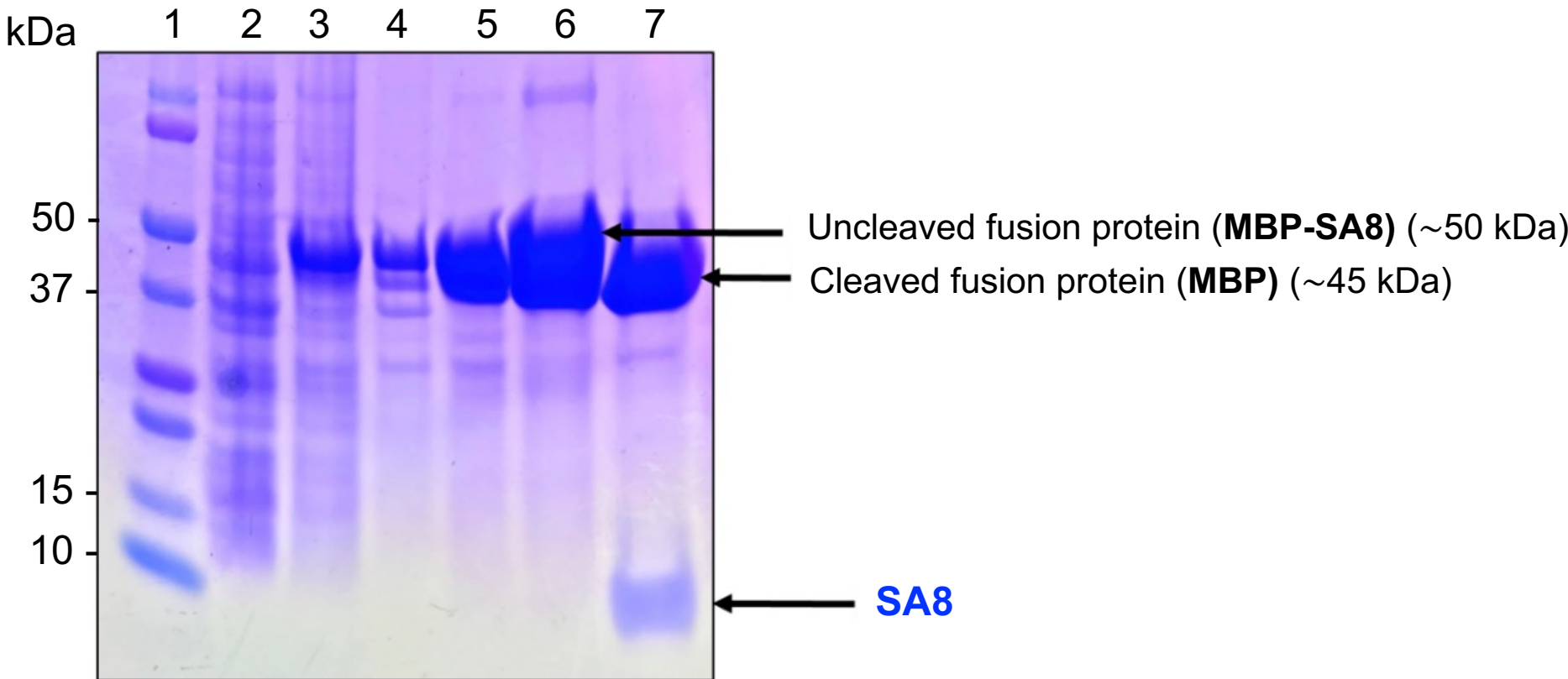

Supplement: Supplementary file 5 — Additional file 5: Figure S2. SDS-PAGE gel stained with Coomassie Blue showing samples of the His6-MBP-SA8 fusion protein obtained during different steps of expression and purification. Lane 1: molecular mass standards; Lanes 2 and 3: E. coli cells pre- and post-induction with IPTG; Lane 4: sucrose extract; Lane 5: periplasmic extract; Lanes 6 and 7: purified MBP fusion protein before and after cleavage with TEV protease. [file 12915_2023_1617_MOESM5_ESM.pdf]

**A**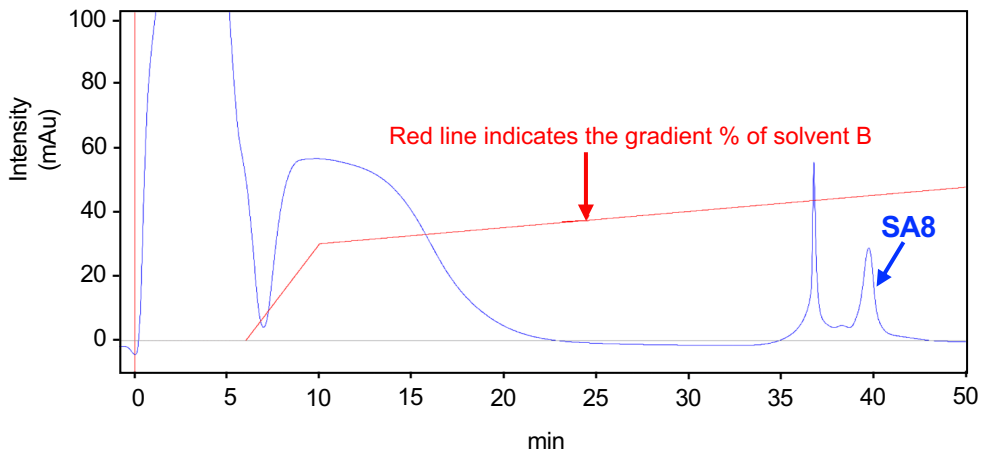**B**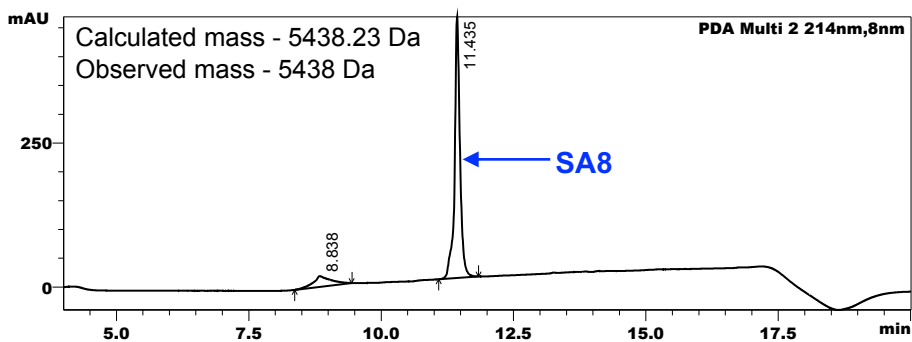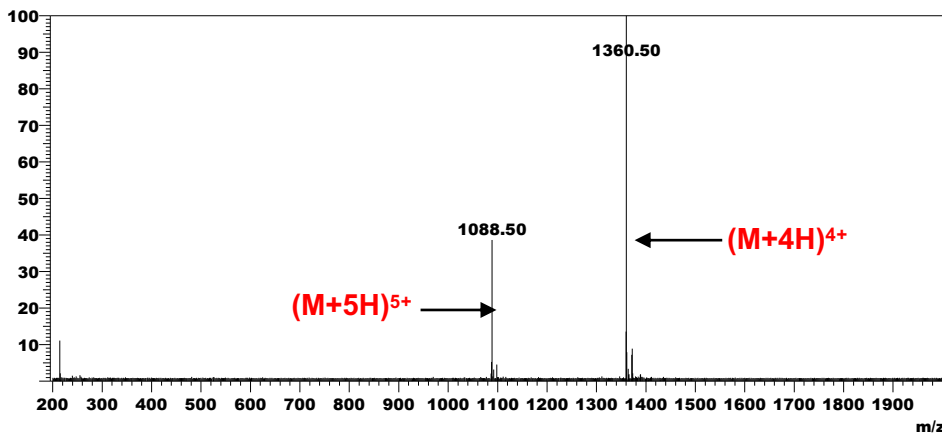

Supplement: Supplementary file 6 — Additional file 6: Figure S3. RP-HPLC purification of recombinant SA8. The peptide was separated on a Vydac C18 columnusing a flow rate of 1 mL/min and a 40 min linear gradient of 30–50% solvent B, as indicated by the red line. LC-MS profile of oxidised SA8 after purification using RP-HPLC. The observed mass is consistent with formation of three disulfide bonds. [file 12915_2023_1617_MOESM6_ESM.pdf]

**A**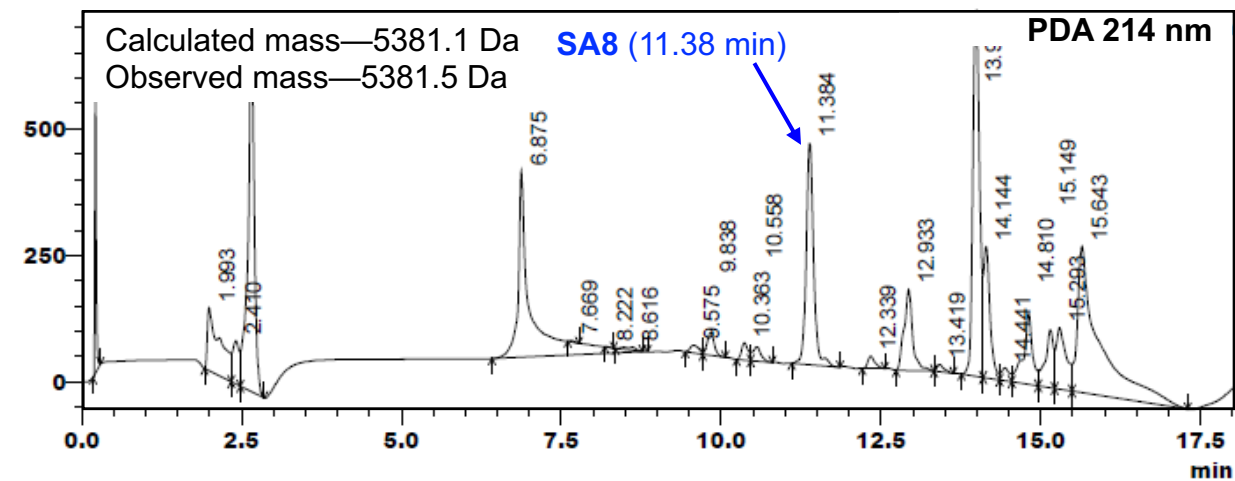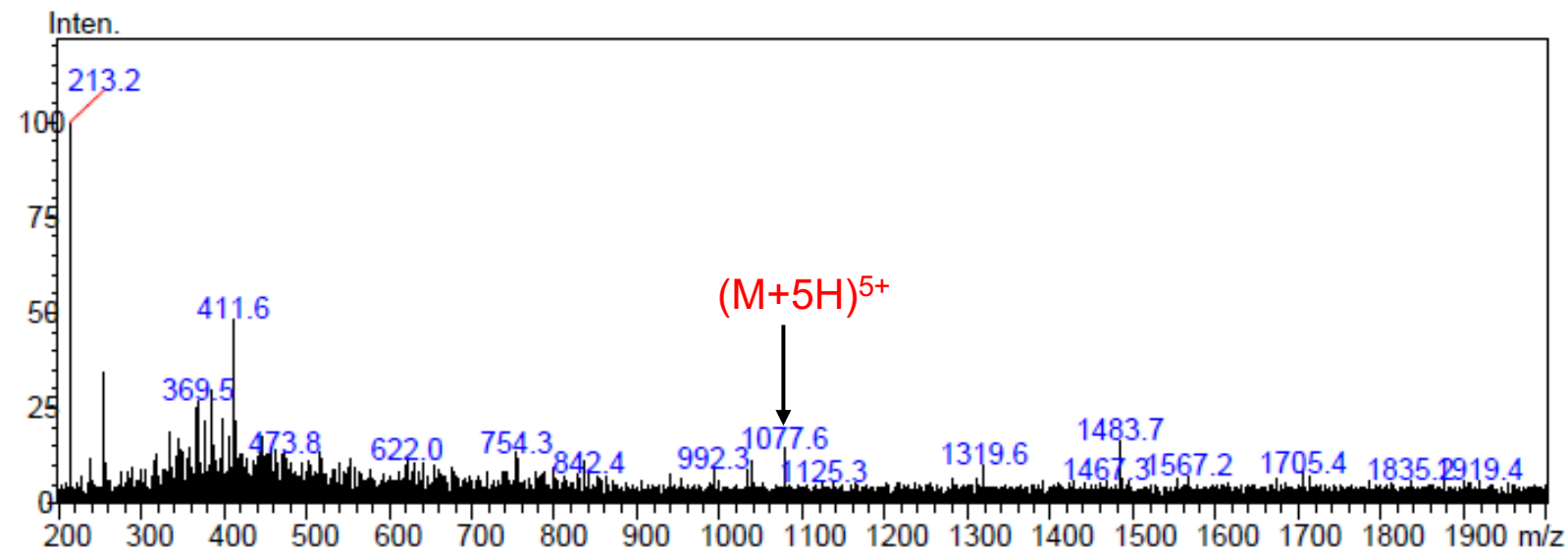**B**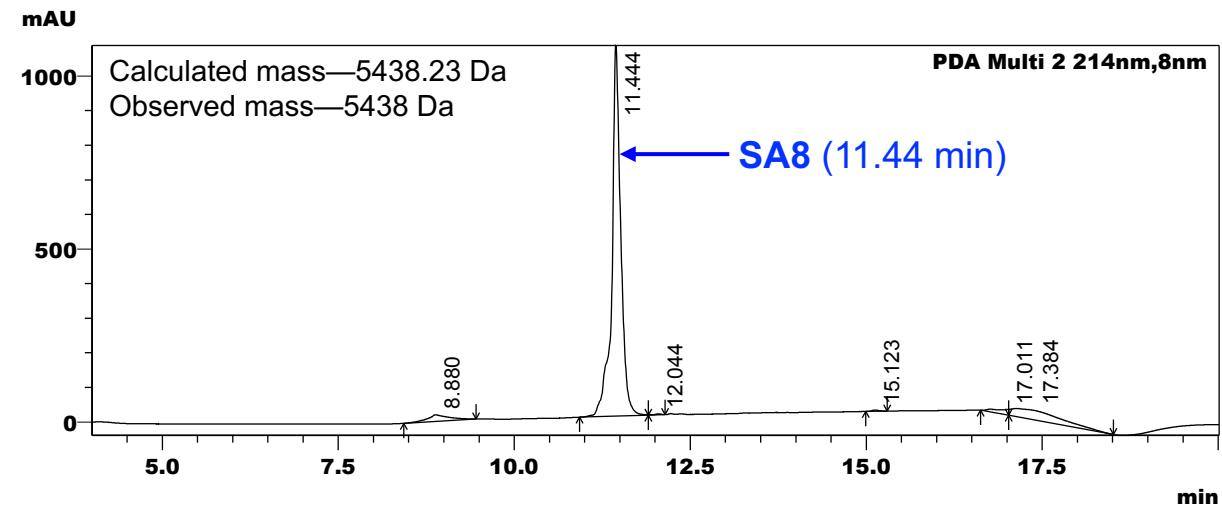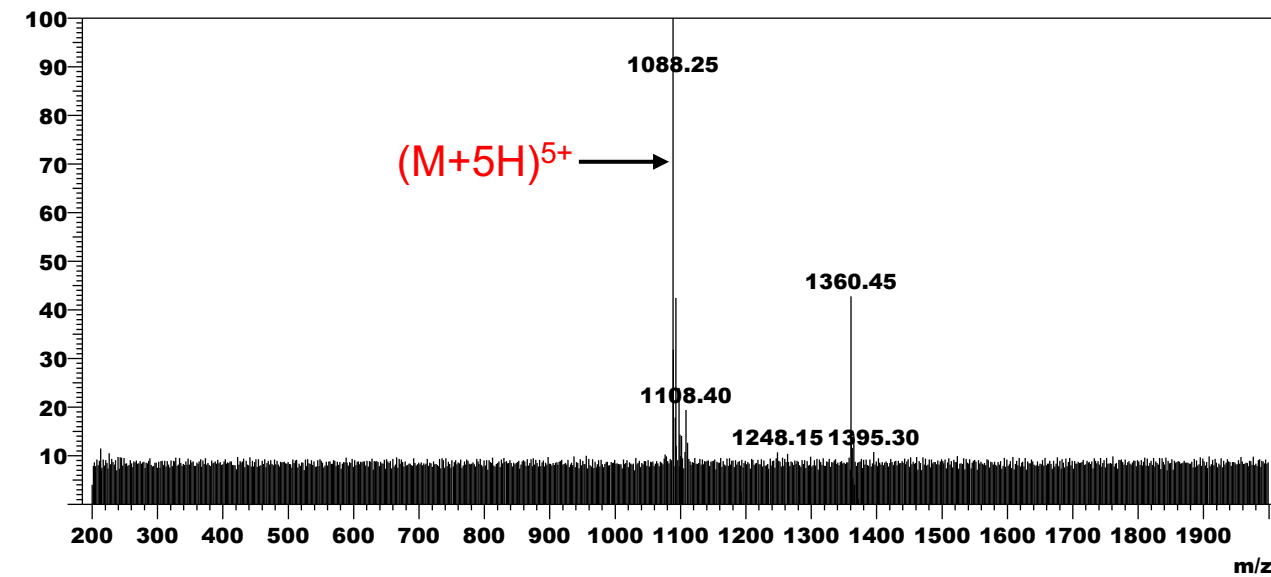

Supplement: Supplementary file 7 — Additional file 7: Figure S4. Comparison of LC-MS profiles for co-eluted native and recombinant SA8. LC-MS profile of the native SA8 peptide. LC-MS profile of the recombinant SA8 peptide. [file 12915_2023_1617_MOESM7_ESM.pdf]

**A**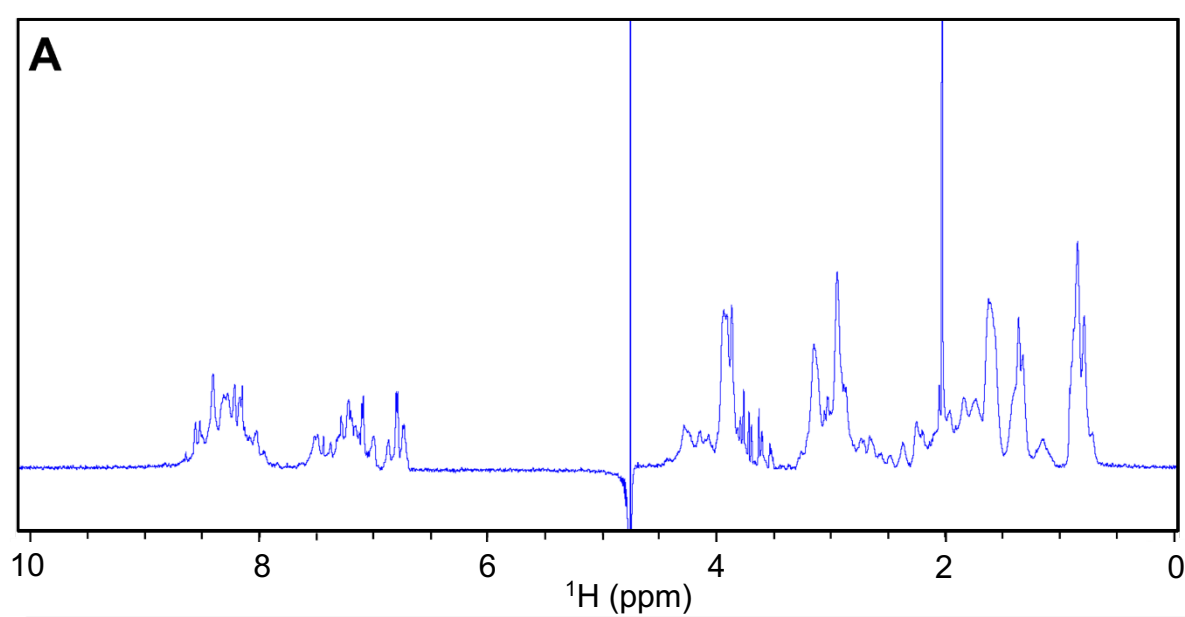**B**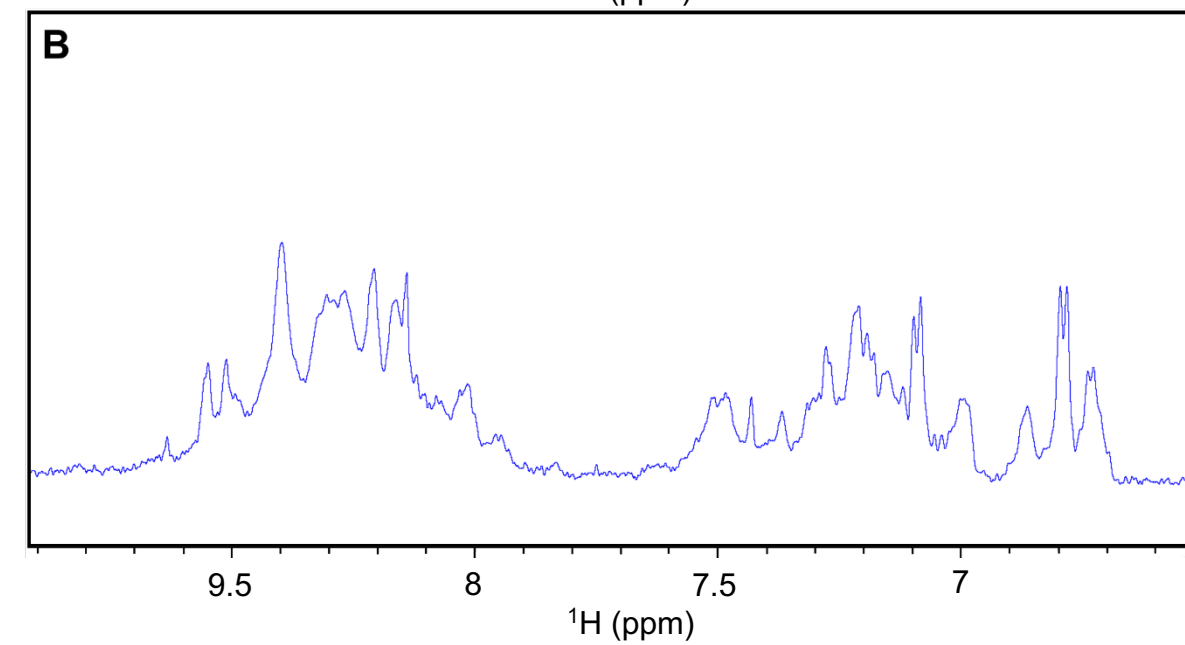

Supplement: Supplementary file 9 — Additional file 9: Figure S5. Full 1D 1H NMR spectrum of recombinant SA8 recorded on Bruker 600 MHz NMR spectrometer at pH 3.5 and 298 K and 256 scans. Expanded amide/aromatic region. [file 12915_2023_1617_MOESM9_ESM.pdf]

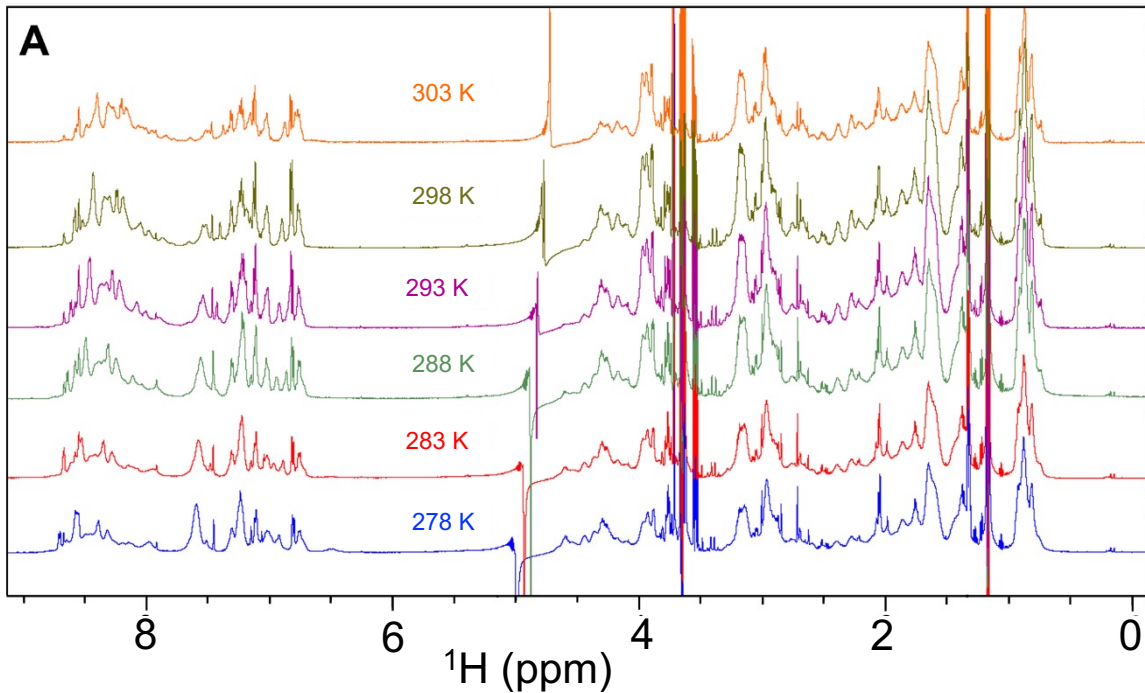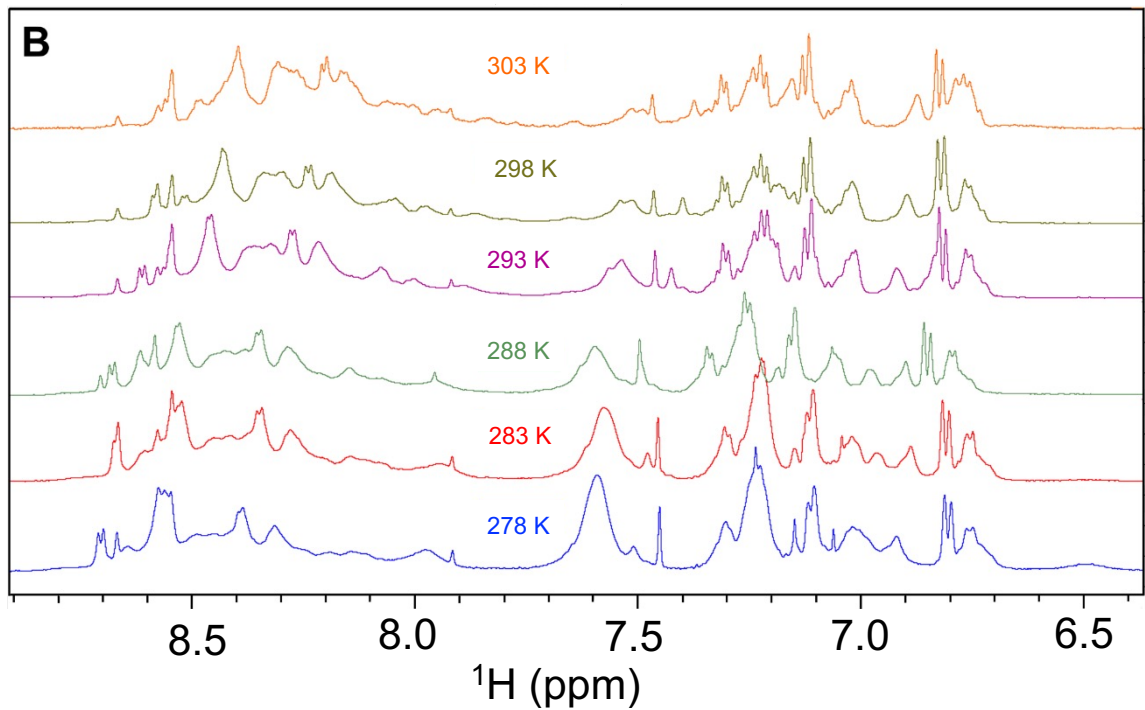

Supplement: Supplementary file 10 — Additional file 10: Figure S6. 1D 1H NMR spectra of SA8 recorded on Bruker 600 MHz NMR spectrometer. Spectra were acquired at pH 3.5 and different temperatures over the range 5–30 °C. Expanded amide/aromatic region. [file 12915_2023_1617_MOESM10_ESM.pdf]

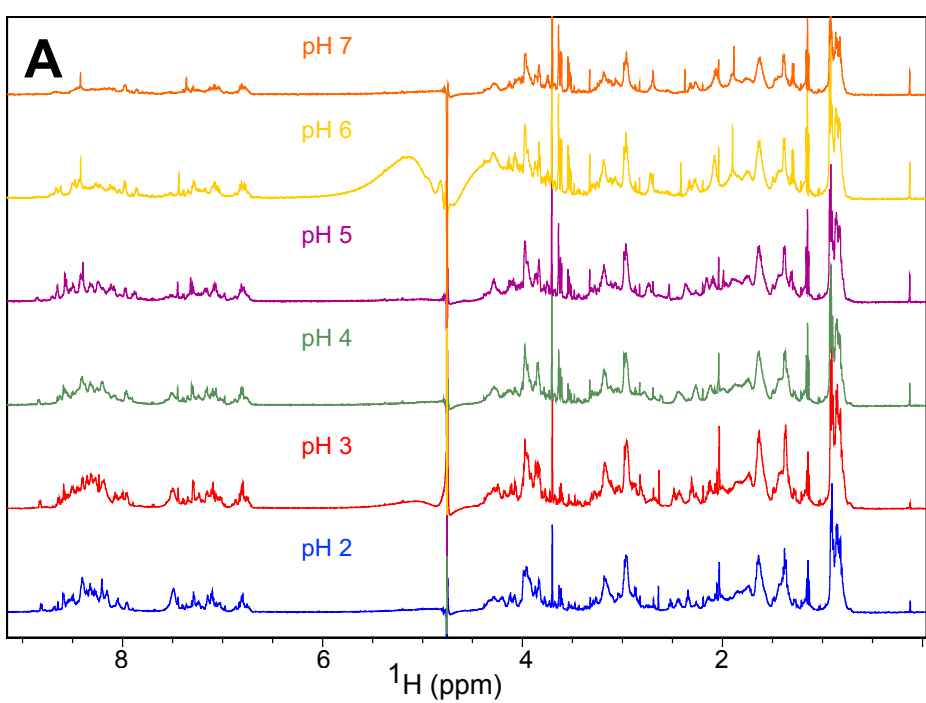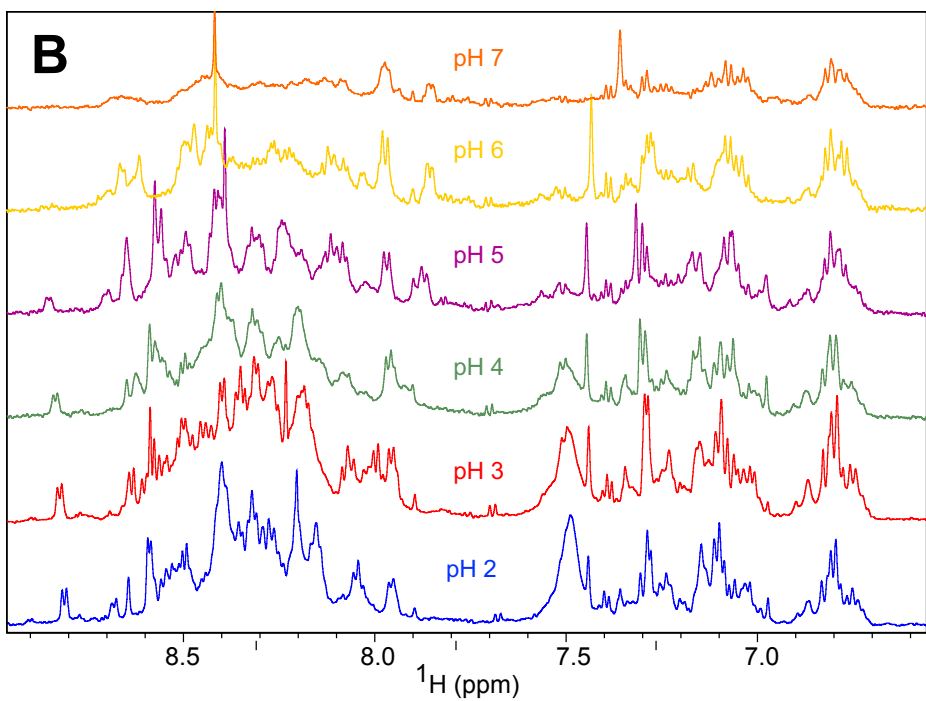

Supplement: Supplementary file 11 — Additional file 11: Figure S7. 1D 1H NMR spectra of SA8 recorded on Bruker 600 MHz NMR spectrometer. Spectra were acquired at 298 K using 256 scans, over the pH range 2-7. Expanded amide/aromatic region. [file 12915_2023_1617_MOESM11_ESM.pdf]

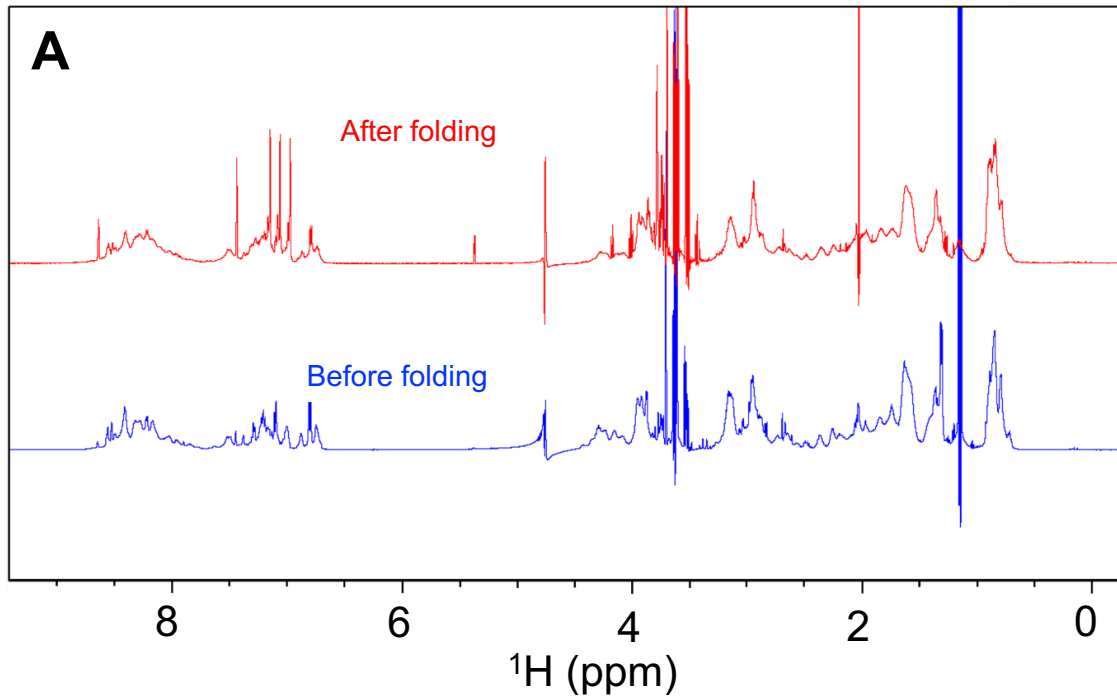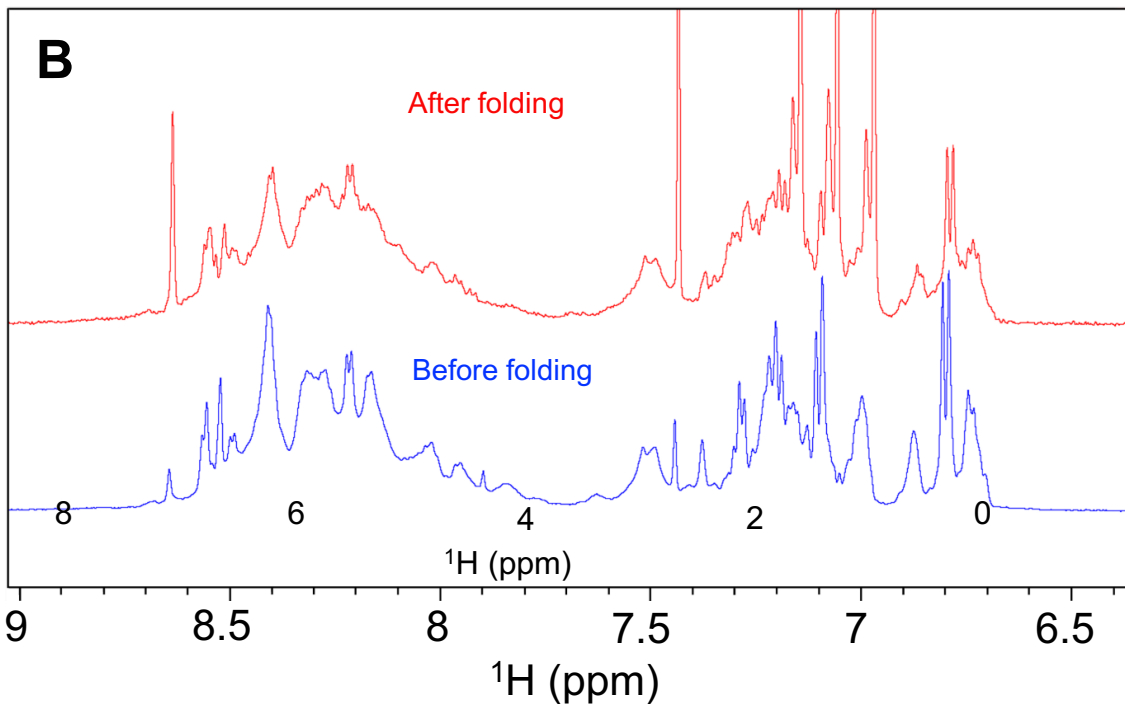

Supplement: Supplementary file 12 — Additional file 12: Figure S8. 1D 1H NMR spectra of SA8 recorded on Bruker 600 MHz NMR spectrometer. Spectra were acquired at pH 3.5 and 298 K using 256 scans. Expanded amide/aromatic region. N.B. The sharp strong peaks in the spectra are from low molecular weight molecules. [file 12915_2023_1617_MOESM12_ESM.pdf]

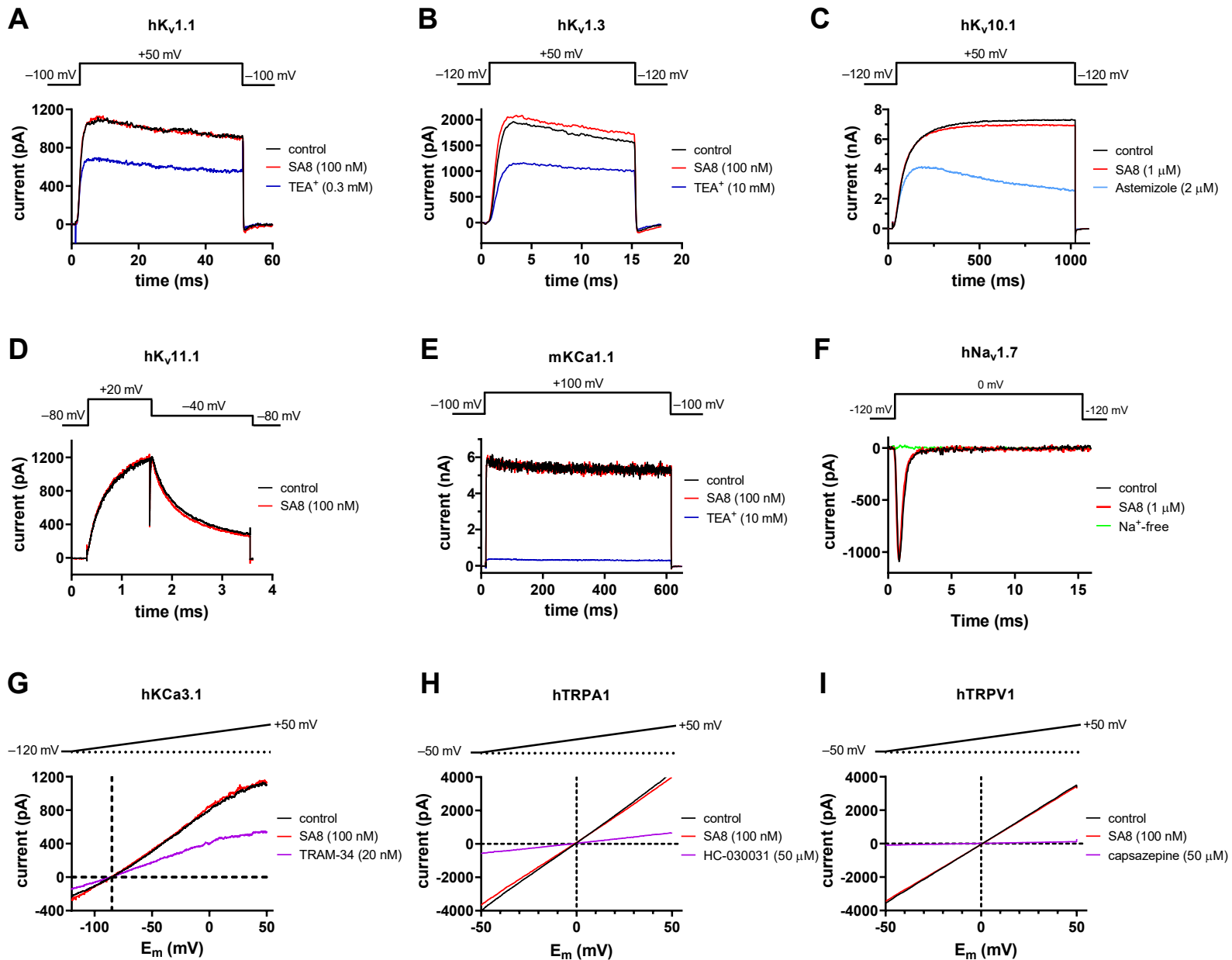

Supplement: Supplementary file 13 — Additional file 13: Figure S9. SA8 has no effect on several voltage-gated and Ca2+-activated ion channels. Current traces recorded before application of SA8, after 1-2 min perfusion with SA8, and after perfusing the recording chamber with control solutions. Data are shown for the following channels: HkV1.1, hKV1.3, hKV10.1, hKV11.1, mKCa1.1, hNaV1.7, hKCa3.1, hTRPA1, and hTRPV1. For details on the expression systems, solutions, and voltage protocols, see Materials and Methods. For hKCa3.1, hTRPA1, and hTRPV1, the currents were recorded in response to a voltage ramp, corrected for ohmic leakage and then displayed as a function of test potential. The horizonal dashed line shows the zero current level and the vertical dashed line indicates the expected reversal potential for K+ . [file 12915_2023_1617_MOESM13_ESM.pdf]
